# Tumor-to-Tumor Metastasis: Lung Carcinoma Metastasizing to Thyroid Neoplasms

**DOI:** 10.1155/2015/153932

**Published:** 2015-01-01

**Authors:** Shiuan-Li Wey, Kuo-Ming Chang

**Affiliations:** Department of Pathology, Mackay Memorial Hospital, Hsinchu 30071, Taiwan

## Abstract

Tumor-to-tumor metastasis is extremely rare in the thyroid glands, and only seven cases of lung carcinoma metastasizing to thyroid tumors have been reported in the literature. We report another two cases of lung carcinoma metastasizing to thyroid neoplasms and review of the literature. The first case was a 64-year-old man presenting with neck mass, hoarseness, and easy choking for 2 months. Image studies showed several nodular lesions within bilateral thyroid glands. A histological examination after radical thyroidectomy revealed lung small cell carcinoma metastasizing to a thyroid follicular adenoma. The second case was a 71-year-old woman with a history of lung adenosquamous carcinoma. The PET/CT scan showed left lower lung cancer and a hypermetabolic area in the right thyroid lobe, highly suspicious for malignancy. Radical thyroidectomy and left lung lobectomy were performed, and the thyroid gland revealed lung adenosquamous carcinoma metastasizing to a papillary thyroid carcinoma.

## 1. Introduction

The thyroid gland is an uncommon site for metastatic tumors, and most thyroid gland tumors are primary. The overall incidence of metastatic thyroid gland tumors is ranging from 1.4 to 3% [1]. Tumor-to-tumor metastasis is exceedingly rare, which is defined as the recipient tumor being a true neoplasm and the donor neoplasm being a true metastasis [2, 3]. To our knowledge, only seven cases of lung carcinoma metastasis to thyroid gland tumors have been reported in the literature [2–8]. Among histological types of lung carcinoma metastasizing to thyroid neoplasms, adenocarcinoma was the most commonly reported, followed by poorly differentiated carcinoma and small cell carcinoma. We present one case of lung small cell carcinoma metastasizing to thyroid follicular adenoma and another case of lung adenosquamous carcinoma metastasizing to papillary thyroid carcinoma.

## 2. Case Presentation

### 2.1. Case 1

**Figure 1:**
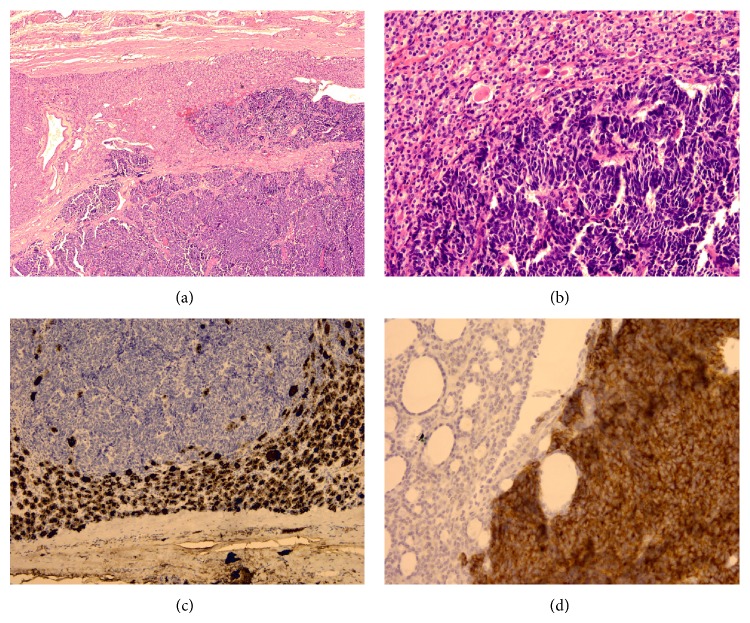
(a) Within the encapsulated follicular adenoma is an abrupt transition to a morphologically distinct neoplasm (magnification ×40). (b) Metastatic carcinoma is arranged in sheets with hyperchromatic nuclei and scanty cytoplasm infiltrating the follicular adenoma (magnification ×200). (c) The metastatic carcinoma is negative for thyroglobulin, while the adenoma is strongly positive (magnification ×100). (d) Synaptophysin is strongly positive in the metastatic carcinoma (magnification ×200).

A 64-year-old man, who had no past history of major disease, presented with neck mass, hoarseness, and easy choking. Fine needle aspiration of bilateral thyroid glands was performed and cytology showed plenty of single or cohesive tumor cells, and anaplastic carcinoma was suspected. Image studies revealed several nodular lesions with strong heterogeneous enhancement within bilateral thyroid glands. The patient received a radical thyroidectomy. On gross examination, the left thyroid gland was 7.3 × 4 × 3.5 cm in size with one major well-defined nodule, and the right thyroid gland was 5.5 × 4 × 3 cm in size with several nodules. The major well-defined nodule in the left thyroid gland measured 4.5 × 3.5 × 3 cm in size, which appeared tan and partially white in color. It was solid and soft to mildly firm in consistency. Histological exam of thyroid glands revealed follicular adenoma with thick to thin capsule in left side and adenomatoid nodules in right side. Within follicular adenoma and adenomatoid nodules were multifocal areas showing an abrupt transition to a morphologically distinct neoplasm comprised of cells with hyperchromatic nuclei and scanty cytoplasm arranged in large sheets and broad ribbons (Figures 1(a) and 1(b)). The immunohistochemical stains of those neoplastic cells with hyperchromatic nuclei were positive for synaptophysin, chromogranin-A, TTF-1, and cytokeratin, while being negative for calcitonin and thyroglobulin (Figures 1(c) and 1(d)). The surrounding nonneoplastic gland was not involved by the carcinoma. The features suggest metastatic small cell carcinoma of lung origin. Postoperative computed tomography revealed a 4.7 cm tumor mass in the left upper lobe of lung with mediastinal lymphadenopathy. A mediastinum lymph node biopsy was performed. There were many metastatic neoplastic cells present in the lymph node which cytomorphologically and immunohistochemically was identical to metastatic small cell carcinoma in thyroid glands. Small cell carcinoma of lung metastasizing to follicular adenoma and adenomatoid nodules of thyroid glands was diagnosed.

### 2.2. Case 2

**Figure 2:**
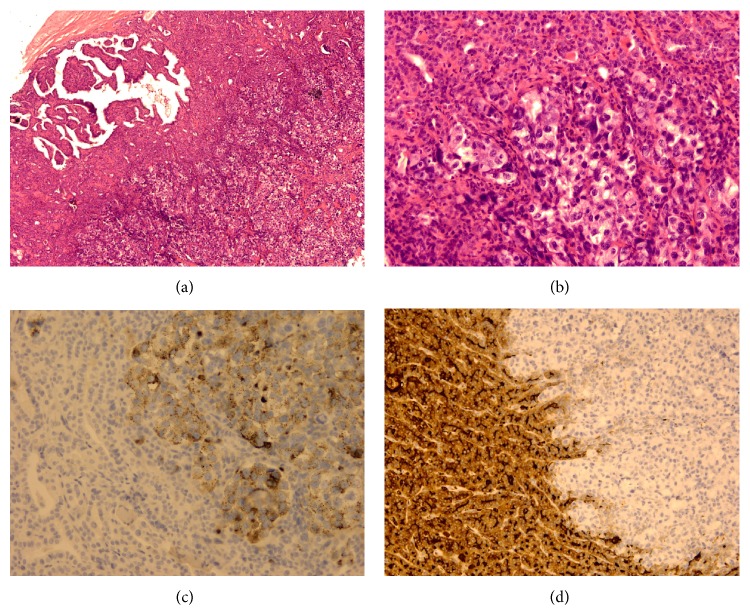
(a) Within the papillary carcinoma is an abrupt transition to a morphologically distinct neoplasm (magnification ×40). (b) Metastatic carcinoma is arranged in nests with large nuclei, nucleoli, and abundant clear-to-eosinophilic cytoplasm (magnification ×200). (c) Napsin-A is shown to be positive in the metastatic carcinoma (magnification ×200). (d) Thyroglobulin is negative in the metastatic carcinoma, while being strongly positive in papillary carcinoma (magnification ×100).

A 71-year-old woman presented with a pulmonary mass diagnosed as adenosquamous carcinoma based on biopsy and received adjuvant chemotherapy. Three months later, PET/CT scan revealed left lower lung cancer and a hypermetabolic area in the right thyroid lobe, highly suspicious for malignancy. Fine needle aspiration of right thyroid gland was performed and cytology showed groups of follicular cells with features of papillary thyroid carcinoma. The patient received a radical thyroidectomy and left lung lobectomy. On gross examination, there was a tumor measuring 5.6 × 4.5 × 3 cm in size in the left lower lobe of lung, and the histological exam revealed adenosquamous carcinoma. The right thyroid gland was 4.4 × 2.3 × 1.8 cm in size with one major nodule and several small nodules. Histological exam of the major nodule revealed papillary thyroid carcinoma. Within the nodule was a focal area showing an abrupt transition to a morphologically distinct neoplasm comprised of cells with large nuclei, distinctive nucleoli, and abundant eosinophilic cytoplasm arranged in solid nests and few glandular pattern (Figures 2(a) and 2(b)). The mucicarmine stain reveals cytoplasmic positive in those large neoplastic cells. The immunohistochemical stains of large neoplastic cells were also positive for TTF-1 and napsin-A, while being negative for thyroglobulin (Figures 2(c) and 2(d)). A part of adenocarcinoma of primary lung adenosquamous carcinoma metastasizing to a papillary thyroid carcinoma was diagnosed.

## 3. Discussion

Metastatic disease to thyroid gland is uncommon, with reported incidence ranging from 1.4 to 3% of all patients who undergo surgery for suspected cancer in the thyroid gland [1]. The pathogenesis of metastasis is either through lymphovascular spread or extension from adjacent tissue [9]. Although the thyroid glands are rich in blood supply, few metastatic tumors occur. Fast arterial flow, high oxygen saturation, and iodine content of the thyroid glands are inhibiting the growth of malignant cells [1]. The most common metastatic tumors in thyroid were from the kidney, lung, gastrointestinal tract, and breast [1, 9]. Lung carcinoma was the second common neoplasm to metastasize to the thyroid gland. Among histological types of lung carcinoma, adenocarcinoma was the most commonly reported tumor, followed by squamous cell carcinoma, large cell carcinoma, and small cell carcinoma [10]. In the study of Chung et al., 44.2% metastatic tumors to the thyroid gland occurred in glands with abnormalities, such as neoplasm or benign conditions [1]. The abnormal thyroid gland may have decreased blood supply resulting in decreased oxygen content and iodine content and thus is more vulnerable to metastatic malignancy [1, 3, 11].

Metastasis to a thyroid neoplasm—tumor-to-tumor metastasis—is extremely rare, and only about 31 cases have been reported in the literature [3, 5, 7, 11]. Tumor-to-tumor metastasis is defined as the recipient tumor being a true neoplasm and the donor neoplasm being a true metastasis [3]. Renal cell carcinoma was the most common primary tumor metastasizing to a thyroid neoplasm (10 cases), followed by lung carcinoma (7 cases), breast carcinoma (5 cases), colon carcinoma (3 cases), and others [3, 5, 8, 11]. We presented 2 cases of lung carcinoma metastasizing to thyroid tumors.

In the 9 cases of lung carcinoma metastasizing to thyroid tumor (including our 2 cases, Table 1), the recipient tumor was follicular adenoma in 5 cases, follicular variant of papillary thyroid carcinoma in 3 cases, and papillary carcinoma in 1 case [2–8]. The donor tumor was adenocarcinoma in 4 cases [4–6, 8], poorly differentiated carcinoma in 2 cases [3, 7], small cell carcinoma in 2 cases [2], and adenosquamous carcinoma in 1 case. The mean age of these patients was 63 (46–75) years old, and the male to female ratio was 4 : 5. Diagnosis of primary tumor and its metastasis into a thyroid neoplasm were synchronous in two case, metachronous in four cases (2 months–2 years), and autopsy in three cases. We presented the first case of metastatic lung adenosquamous carcinoma in papillary thyroid carcinoma and another case of metastatic small cell carcinoma in thyroid follicular adenoma.

Tumor-to-tumor metastasis should be considered when a distinct histological pattern is encountered in a tumor or in a patient with previous history of malignancy. Preoperative diagnosis of a primary thyroid tumor versus metastatic disease is difficult because of similar radiological findings and clinical presentations [11]. The history of previous lung carcinoma may be helpful, and fine needle aspiration cytology of tumor is also useful. However, two of our cases were misinterpreted as a primary thyroid tumor in fine needle aspiration cytology due to lack of clinical history and sampling error. Hence, the diagnosis of metastatic lung carcinoma was made with histological examination and immunohistochemical studies after thyroidectomy. To distinguish metastatic malignancy from a primary thyroid neoplasm on histology or cytology, this may require further immunohistochemical or molecular studies [9]. The thyroid tumors are usually reactive for immunohistochemical markers of thyroglobulin and thyroid transcription factor-1 (TTF-1). However, most of the lung adenocarcinomas and small cell carcinomas are also positive for TTF-1, making the distinction even more challenging. More specific markers are needed for differential diagnosis, such as neuroendocrine markers and mucicarmine stain. Molecular studies and a dual-probe “break-apart” fluorescence* in situ* hybridization (FISH) assay are also helpful in the diagnosis of thyroid papillary carcinoma and lung carcinomas.* RET/PTC* rearrangements,* BRAF* mutations, and* RAS* mutations are frequently identified in thyroid papillary carcinoma, while* EGFR* mutations and* ALK* rearrangements are usually found in lung carcinoma [12].

The treatment of metastatic disease is dependent on the stage and grade of the primary tumor, extension of the thyroid lesion, and the general condition of the patient [13]. Thyroidectomy is generally performed in the patients with minimal disease in the thyroid and no evidence of metastasis in other sites. In these patients, the prognosis is good. Surgery is also palliative for relieving compressive symptoms in patients with disseminated disease [11, 13]. The extent of surgery should depend on the ability to completely remove the metastatic tumor.

In conclusion, tumor-to-tumor metastasis in thyroid gland is exceedingly rare, and it should be considered in patients with a thyroid mass and previous history of malignancy. The abnormal thyroid glands with goiter or tumors might be more susceptible to metastatic malignancies because of a decrease in oxygen and iodine content. Lung carcinoma is the second common primary tumor metastasizing to a thyroid neoplasm, and only about 7 cases have been reported in the literature. We present another two cases of lung carcinoma metastasizing to thyroid tumors. The distinction between primary and metastatic tumors is difficult in some cases. Careful histological examination and molecular and immunohistochemical studies are helpful for differential diagnosis.

## Figures and Tables

**Table 1:** Lung carcinoma metastasizing to thyroid tumor: the cases reported in the literature.

Authors	Age	Gender	Receiving thyroid neoplasm	Lung carcinoma	Interval
Akamatsu et al. [4]	46	Female	Follicular adenoma	Well-differentiated adenocarcinoma	4 months
Hashimoto et al. [5]	60	Female	Follicular variant of papillary thyroid carcinoma	Adenocarcinoma	Synchronous
Kameyama et al. [6]	51	Male	Follicular adenoma	Moderately differentiated adenocarcinoma	Autopsy
Mori et al. [8]	54	Male	Follicular variant of papillary thyroid carcinoma	Poorly differentiated adenocarcinoma	Autopsy
Stevens et al. [3]	65	Male	Follicular adenoma	Poorly differentiated carcinoma	2 months
Mizukami et al. [7]	75	Female	Follicular adenoma	Poorly differentiated carcinoma	Autopsy
Baloch and LiVolsi [2]	75	Female	Follicular variant of papillary thyroid carcinoma	Small cell carcinoma	2 years
Wey (present case 1)	66	Male	Follicular adenoma	Small cell carcinoma	Synchronous
Wey (present case 2)	72	Female	Papillary thyroid carcinoma	Adenosquamous carcinoma	3 months
